# Effects of Subthalamic Nucleus Deep Brain Stimulation on Depression in Patients with Parkinson’s Disease

**DOI:** 10.3390/jcm11195844

**Published:** 2022-10-01

**Authors:** Tianqi Hu, Hutao Xie, Yu Diao, Houyou Fan, Delong Wu, Yifei Gan, Fangang Meng, Yutong Bai, Jianguo Zhang

**Affiliations:** 1 Department of Neurosurgery, Beijing Tiantan Hospital, Capital Medical University, Beijing 100070, China; 2 Beijing Key Laboratory of Neurostimulation, Beijing 100070, China

**Keywords:** Parkinson’s disease, subthalamic nucleus, deep brain stimulation, depression

## Abstract

Objective: In this study, we aimed to investigate the effects of STN-DBS on PD patients with different levels of depression and to identify predictors of the effects of STN-DBS on PD depression. Methods: We retrospectively collected data for 118 patients with PD depression who underwent STN-DBS at Beijing Tiantan Hospital. Neuropsychological, motor, and quality of life assessments were applied preoperatively and postoperatively. All patients were divided into two groups according to their HAM-D_24_ total scores (group I: mild depression; group Ⅱ: moderate depression). A mixed repeated-measure analysis of variance (ANOVA) was performed to investigate whether there were differences in depression scores before and after STN-DBS between the two groups. The changes in depression scores were also compared between groups using ANCOVA, adjusting for gender and preoperative HAMA scores. Logistic regression was performed to identify predictors of STN-DBS’s effects on PD depression. Results: Both groups showed significant improvement in depression symptoms after STN-DBS. Compared with patients in group I, patients in group Ⅱ showed greater reductions in their HAM-D_24_ total scores (*p* = 0.002) and in HAM-D_24_ subitems including cognitive disturbances (*p* = 0.026) and hopelessness symptoms (*p* = 0.018). Logistic regression indicated that gender (female) (*p* = 0.014) and preoperative moderate depression (*p* < 0.001) patients had greater improvements in depression after STN-DBS. Conclusions: Patients with moderate depression showed better improvement than patients with mild depression. Gender (female) and preoperative HAMA scores are predictors of STN-DBS’s effects on PD depression.

## 1. Introduction

Parkinson’s disease (PD) is a neurodegenerative disorder characterized by motor symptoms (tremor, rigidity, and slowness of movements) and several non-motor symptoms [1,2,3,4]. Depression, one of the most common non-motor symptoms, is a chronic and commonly reported neuropsychiatric disorder that occurs in 40%–50% of patients with PD [5,6]. PD depression is mainly described as feelings of worthlessness or guilt, a depressed mood, reversible dementia, loss of interest in daily activities, recurrent suicidal tendencies [7,8,9,10], and somatic complaints (muscle tension or sexual dysfunction) [11]. Symptoms of depression can occur at the initial stage of PD motor symptoms and can worsen over time [12], thus negatively affecting quality of life.

To date, several new techniques, for example, deep brain stimulation (DBS), transcranial magnetic stimulation (TMS), and transcranial direct current stimulation (tDCS), are being used for the treatment of PD. DBS is a widely used surgical technique that involves the implantation of multiple-contact electrodes in specific brain regions. Targets of DBS for the treatment of PD include the subthalamic nucleus (STN), external globus pallidus (GPi), pedunculopontine nucleus (PPN), and the substantia nigra pars reticulata (SNr) [13]. Subthalamic nucleus deep brain stimulation (STN-DBS) is a well-established surgical treatment for PD. Although STN-DBS is widely acknowledged to control motor symptoms, including tremor, rigidity, and bradykinesia, the efficacy of STN-DBS in treating PD depression remains controversial. Reports have shown that STN-DBS significantly ameliorates symptoms of depression post-operatively, and its efficacy is greater 6 months after the operation [14,15]. Some articles have concluded that STN-DBS shows little evidence of ameliorating depression, whereas Follett et al. have reported the worsening of depressive disorder after STN-DBS [16]. In another article, suicidal behavior was found to occur within 3 years postoperatively, and the suicide rate was elevated in patients with severe depression [17], thus suggesting that patients with severe depression are not candidates for surgery. Furthermore, the authors of one study declared that PD patients become angry more easily after STN-DBS [18].

Several factors may explain the reported differences in the efficacy of STN-DBS for PD depression. First, the participants in the experiments might have had different baseline data (varying degrees of depression). Moreover, differences in study design, assessment conditions (on medication vs. off medication), and different stimulator settings might have led to contradictory results. However, despite these possibilities, the factors associated with changes in postoperative depression remain unclear. Therefore, our study aimed at verifying the exact effects of STN-DBS on PD depression by dividing patients with PD into groups according to the severity of their depression and further investigating possible predictive factors of STN-DBS’s efficacy in treating PD depression.

## 2. Methods

### 2.1. Patient Selection

We retrospectively collected data from patients at Beijing Tiantan Hospital (Beijing, China) from January 2016 to August 2021. The inclusion criteria were as follows: (1) diagnosis of idiopathic PD according to the United Kingdom Brain Bank criteria [1]; (2) successful performance of bilateral STN-DBS; (3) performance of motor and psychological assessments (preoperative and postoperative) with available postoperative follow-up ranging from 6 to 12 months; (4) Mini-Mental State Examination (MMSE) score > 24; and (5) all patients suffered from depressive symptoms with HAM-D_24_ scores > 0. The exclusion criteria were as follows: (1) postoperative complications, such as cerebral hemorrhage and hemiplegia; (2) clear brain structural abnormalities, intracranial tumors, or prior brain surgery other than STN-DBS; (3) revised or replaced DBS leads; and (4) incomplete assessment data; and (5) patients with a total HAM-D_24_ score ≥ 35, since major depression is considered a contraindication for STN-DBS. This study was conducted under the approval of the IRB of Beijing Tiantan Hospital. All patients provided written informed consent for their participation.

### 2.2. DBS Electrode Implantation

The standard surgical procedure was as previously described [19,20]. Briefly, quadripolar DBS electrodes (model 3389, Medtronic, Minneapolis, MN, USA, or model L301, Pins Medical, Beijing, China) were implanted under the guidance of a Leksell microstereotactic system (Elekta Instrument AB, Stockholm, Sweden) with pre-surgical MRI under local anesthesia. Intra-operative single-unit recordings and high-frequency stimulation testing were performed to evaluate the optimal locations for permanent electrode implantation. The STN target coordinates for the lower contact were 2–3 mm posterior to the MCP, 12–14 mm lateral to the AC-PC, and 4–6 mm below the inter-commissural line. The electrodes were then connected to an implantable pulse generator implanted in the subclavicular area in patients under general anesthesia. We performed post-operative CT to exclude intracranial hemorrhage, then merged the images with the preoperative MR images to verify the exact locations of the electrodes.

### 2.3. Clinical Assessment

The age, sex, time of onset, duration of disease, and Hoehn and Yahr scale results were recorded for all patients in detail. The levodopa-equivalent daily dose (LEDD) for all patients is presented to provide a better understanding of the dosage of anti-Parkinson’s disease drugs. The proportions of PD patients taking antidepressants preoperatively and postoperatively were also recorded. The Unified Parkinson’s Disease Rating Scale part Ⅲ (UPDRS-Ⅲ) or Movement Disorder Society Unified Parkinson’s Disease Rating Scale part Ⅲ (MDS-UPDRS part Ⅲ) was applied to all patients to evaluate their motor symptoms. Other related symptom scales were used, including the Hamilton Rating Scale for Anxiety (HAMA), Parkinson’s Disease Questionnaire (PDQ-39), and MMSE. PDQ-39 is the most thoroughly validated and extensively used scale assessing the quality of life among patients with PD [21,22], and the MMSE scale is used to assess cognitive function. Motor assessments were applied in two conditions (off medication and on medication) 1 week before surgery preoperatively, and two conditions (off medication/on stimulation and on medication/on stimulation) postoperatively. MDS-UPDRS-Ⅲ improvement was calculated as (pre-MDS-UPDRS-Ⅲ [med-off]–post-MDS-UPDRS-Ⅲ [stim on/med off])/pre-MDS-UPDRS-Ⅲ [med-off] ×100%. We selected the assessment results of the last return to the hospital for follow-up analysis (at least 6 months postoperatively). All assessments were conducted by a movement disorder specialist in our center, and all motor evaluations were video-recorded. Off-medication assessment was performed at least 12 h after withdrawal from dopaminergic medications, and on-medication assessment was performed 1 h after medication administration. Post-operative CT was performed at 1 month to verify the exact locations of the electrodes by merging the images with the preoperative MR images and was used to guide electrode choice during the adjustment of stimulation parameters. At each assessment time point, patients were required to visit the outpatient programming service to ensure that all assessments were conducted under optimal stimulation parameters.

We used the 24-item Hamilton depression rating scale (HAM-D_24_) as a measurement of the severity of depression [23]. The HAM-D_24_ contains 24 items, with 10 items ranging from 0–2 and 14 items ranging from 0–4. Items ranging from 0–2 points were defined as follows: 0: none; 1: mild to moderate; 2: severe. Items of 0–4 points were defined as follows: 0: none; 1: mild; 2: moderate; 3: severe; 4: very severe. The total scores of HAM-D_24_ ranged from 0 (minimal) to 76 (maximal). The severity ranges for the HAM-D_24_ are divided into four levels: non depression (0–7), mild depression (8–20), moderate depression (21–34), and severe depression (35–76) [24,25,26,27,28] Patients with a total HAM-D_24_ score of 8–20 were assigned to group I (mild depression), whereas those with a total HAM-D_24_ score of 21–34 were assigned to group Ⅱ (moderate depression). Patients with a total HAM-D_24_ score ≥ 35 were excluded, because major depression is considered a contraindication for STN-DBS [29].

To further explore the effects of STN-DBS on different subtypes of depression, we sorted the 24 items of HAM-D_24_ into the following seven subitems, as previously described [30,31]: (1) anxiety/somatization (six items: psychic anxiety, somatic anxiety, gastrointestinal symptoms, hypochondriasis, insight, and general symptoms), (2) weight loss (one item), (3) cognitive disturbances (six items: self-guilt, suicide, agitation, depersonalization and derealization, and paranoid and obsessive-compulsive symptoms), (4) circadian fluctuations (one item), (5) retardation symptoms (four items: depression, work and interests, retardation and sexual symptoms), (6) sleep disturbances (three items: difficulty in falling asleep, superficial sleep and early awakening), and (7) hopelessness symptoms (three items: helplessness, hopelessness, and worthlessness).

### 2.4. Statistical Analysis

All continuous variables are presented as mean ± SD, and categorical variables are presented as percentages. Kolmogorov–Smirnov tests were conducted to determine whether continuous variables were normally distributed. Differences in demographic characteristics were compared between group I (mild depression) and group Ⅱ (moderate depression) using independent *t*-tests, nonparametric tests, and chi-squared tests where appropriate.

To investigate whether there were differences in the severity of depression after STN-DBS between the two groups, a mixed repeated-measure analysis of variance (ANOVA) was performed, with group (group I and group Ⅱ) as the between-group factor and time (before and after DBS) as the within-group factor, followed by Bonferroni’s post hoc tests.

Then, the change in the severity of depression (HAM-D_24_ total scores and subitems) between the preoperative and postoperative periods was calculated for each participant and compared between groups. Comparisons were analyzed first without covariates, using an independent *t*-test. Second, an analysis of covariance (ANCOVA) was conducted to detect differences in the change in depression scores between the preoperative and postoperative periods, while adjusting for covariates. Covariates of gender and preoperative HAMA scores were selected due to their association with depression scores. Apart from the change in depression scores, comparisons were also made with the changes in motor symptoms (MDS-UPDRS-Ⅲ), cognition (MMSE), and quality of life (PDQ-39).

Finally, for differences between groups, categorical variables were compared using the chi-squared test. We applied independent-samples *t*-tests for between-group comparisons when data were normally distributed and nonparametric tests (Mann–Whitney U test) when the data had a skewed distribution. Independent predictors of depression amelioration were analyzed with logistic regression, and family-wise error comparison was also applied for multiple comparisons. Two-tailed *p*-values below 0.05 were considered to indicate significant results. All statistical analyses were conducted in SPSS 24 (IBM, Chicago, IL, USA).

## 3. Results

### 3.1. Patients and Baseline Characteristics

Data for 146 patients with PD were briefly reviewed, and 22 patients were excluded on the basis of the inclusion criteria. Among the 124 patients who met the criteria, two patients were lost to the 12-month follow-up postoperatively, one patient had missing or incomplete psychological evaluation data, and three patients had an accident (trauma or cardiovascular or cerebrovascular disease). The 118 patients who met the criteria were included. The shortest postoperative evaluation time point was 6 months and the longest was 12 months, with an average of 9.78 months in group I (9.78 ± 1.77 months) and 9.88 months in group Ⅱ (9.88 ± 1.68 months). No significant difference was found in the postoperative follow-up between the two groups. For the evaluation of motor symptoms, 42 of 118 patients were evaluated with the UPDRS-III scale, whereas the rest were evaluated with the MDS-UPDRS scale. We conducted score conversion as described previously [32] to transform the UPDRS-III scale into the MDS-UPDRS scale. According to the HAM-D_24_ scale, 78 of the 118 patients were assigned to group I, and 40 patients were assigned to group Ⅱ. For baseline data in the two groups, female patients had more severe depression symptoms than male patients. Patients in group I had lower HAM-D_24_ and HAMA scores than patients in group Ⅱ, and the PDQ-39 scores were also lower in group I than in group Ⅱ, thus indicating that patients in group Ⅱ had more severe depression and lower quality of daily activities. In group I, nine patients (11.54%) were taking antidepressants preoperatively and four patients (6.41%) postoperatively, whereas in group Ⅱ, 10 patients were taking antidepressants preoperatively and six patients postoperatively. No significant differences were found preoperatively (*p* = 0.06) or postoperatively (*p* = 0.14) between the two groups. No other differences were found between groups. Baseline data are shown in Table 1.

### 3.2. Effects of STN-DBS on PD Depression, Motor Symptoms, Cognition, and Quality of Life

The results of the two-way repeated-measures ANOVA are presented in Table 2. The ANOVA revealed a significant main effect of time (F = 66.34, *p* < 0.001), with higher HAM-D_24_ total scores in the preoperative period (18.42 ± 6.51) than in the postoperative period (13.18 ± 8.01), and a main effect of group, with group I being lower (12.71 ± 5.25) than group Ⅱ (20.28 ± 8.77) (F = 118.6, *p* < 0.001). The interaction effect demonstrated that PD patients in group Ⅱ had greater declines in their HAM-D_24_ total scores (F= 12.46, *p* < 0.001) and subitems anxiety/somatization (F = 5.01, *p* = 0.027), cognitive disturbances (F = 10.63, *p* = 0.001) and hopelessness symptoms (F = 4.63, *p*= 0.033). Post hoc analyses indicated that the postoperative HAM-D_24_ total scores were significantly decreased compared to the preoperative period in both group I (*p* < 0.001) and group Ⅱ (*p* < 0.001). For HAM-D_24_ subitems, group I showed significant postoperative declines compared to the preoperative period in anxiety/somatization (*p* = 0.020), circadian fluctuations (*p* = 0.002), retardation symptoms (*p* = 0.012), sleep disturbances (*p* = 0.030), and hopelessness symptoms (*p* = 0.016), whereas group Ⅱ showed significant postoperative declines compared to the preoperative period in anxiety/somatization (*p* < 0.001), cognitive disturbances (*p* < 0.001), circadian fluctuations (*p* = 0.026), retardation symptoms (*p* < 0.001), sleep disturbances (*p* < 0.001), and hopelessness symptoms (*p* < 0.001).

The change between the preoperative and postoperative periods in terms of depression (HAM-D_24_ total score and its subitem scores), as well as motor skills (MDS-UPDRS-Ⅲ score), cognition (MMSE score), and quality of life (PDQ-39 score) are demonstrated in Table 3. The depression levels of a total of 31 patients of the 78 patients (39.74%) in group I decreased from mild (HAM-D_24_ score 8–20) to non-depression (HAM-D_24_ score 1–7). In group Ⅱ, 28 of 40 patients (70%) showed decreased depression levels. Twenty-three patients’ depression levels decreased from moderate (HAM-D_24_ score 21–34) to mild (HAM-D_24_ score 8–20), whereas five patients’ depression levels decreased from mild (HAM-D_24_ score 8–20) to non-depression (HAM-D_24_ score 1–7). Thus, the result of the chi-squared tests showed that the proportion of patients with decreased depression levels after STN-DBS was higher in group Ⅱ than in group I (*p* = 0.002). Independent *t*-tests without covariates indicated that PD patients in group Ⅱ showed greater reductions than patients in group I in terms of the HAM-D_24_ total score (*p* = 0.003) and HAM-D_24_ subitems, including anxiety/somatization (*p* = 0.027), cognitive disturbances (*p* = 0.010), and dopelessness symptoms (*p* = 0.033). Furthermore, adjusting for gender and preoperative HAMA scores resulted in fewer differences between group I and group Ⅱ, with group Ⅱ exhibiting a greater reduction in the HAM-D_24_ total score (*p* = 0.002) and HAM-D_24_ subitems, including cognitive disturbances (*p* = 0.026) and hopelessness symptoms (*p* = 0.018). In addition, the changes between preoperative and postoperative periods in motor symptoms (MDS-UPDRS-Ⅲ), cognition (MMSE), and quality of life (PDQ-39) were also compared between groups; however, no significant differences were found in independent *t*-tests without covariates or ANCOVA adjusting for gender and preoperative HAMA scores. We also calculated the Relative Change as well as Effective Size in both groups. Relative Change= (mean follow-up - mean baseline)/mean baseline. Effective Size= (mean baseline - mean follow-up)/SD change score. The improvement of Anxiety/Somatization, Cognitive Disturbances, Retardation Symptoms, Sleep Disturbances and Hopelessness Symptoms in Group Ⅱ were classified as medium while others as small (Table 4).

### 3.3. Preoperative Predictors of the Effects of STN-DBS on PD Depression

To predict the efficacy of STN-DBS in patients with Parkinson’s disease with different levels of depression in clinical practice, postoperative predictors of Parkinson’s disease depression are necessary. We chose age; sex; age of onset; disease duration; and preoperative factors, including the LEDD, MDS-UPDRS score (med off), HAMA score, HAM-D_24_ score, and PDQ-39 scale score, as possible predictors. After correction, sex (0.35 (0.15, 0.81), *p* = 0.014) and the preoperative HAM-D_24_ score (4.92 (2.00, 12.13), *p* < 0.001) were significant in both univariate and multivariate analyses, thus suggesting that sex and preoperative depression were possible predictors of postoperative depression after STN-DBS (Table 5). PD patients with more severe preoperative depression levels were more likely to gain better effects postoperatively. Female PD patients exhibited better improvements in their depression scores after STN-DBS (0.35 (0.15, 0.81), *p* = 0.014). In contrast, preoperative LEDD and PDQ-39 scores were significant according to the univariate analysis but not according to the multivariate analysis. Other factors were not significant according to both the univariate analysis and multivariate analysis.

## 4. Discussion

In this study, we retrospectively analyzed the effects of STN-DBS on depressive symptoms in PD, as well as the possible preoperative predictors of the effects of STN-DBS on depressive symptoms in PD. STN-DBS significantly decreased HAM-D_24_ scores in patients with PD depression by an average of 10 months after surgery. Interestingly, patients with different levels of depression had different postoperative outcomes. The amelioration of depressive symptoms was greater in patients with moderate depression than in those with mild depression. Thirty-one patients out of 78 patients (39.74%) in group I had decreased depression levels, decreasing from mild (HAM-D_24_ score 8–20) to non-depression, whereas 28 of 40 patients (70%) in group Ⅱ moved from the moderate category (HAM-D_24_ score 21–34) to the mild (HAM-D_24_ score 8–20) or non-depression (HAM-D_24_ score 1–7) categories. PD patients with more severe preoperative depression tended to be more sensitive to stimulation caused by STN-DBS than patients with mild depressive symptoms and had more space for improvement. We speculated that differences in sensitivity to stimulation were responsible for the different results between the two groups. Simultaneously, the major subitem contributors differed among patients with different depression levels. Furthermore, regression analysis on possible preoperative predictors of postoperative depression amelioration indicated that preoperative HAM-D_24_ scores (depression level) and sex predicted the efficacy of STN-DBS in treating depressive symptoms in PD.

In agreement with our results, several recent studies have demonstrated that STN-DBS shows a trend toward the amelioration of PD depression. For example, Huang and colleagues indicated that after STN-DBS, patients’ Beck Depression Inventory scale scores decreased significantly, thus indicating the significant alleviation of depressive symptoms. As previously reported, the generation of the depressive symptoms of PD is associated with the limbic system, the ventromedial prefrontal cortex, and the dorsolateral prefrontal cortex [33,34]. In a resting-state functional MRI (rs-fMRI) study of depression by Wen and colleagues, compared to healthy controls, patients with PD were found to have increased neural activity in prefrontal regions and decreased functional connectivity between prefrontal and limbic structures [35]. Research has demonstrated that STN-DBS influences the connectivity among the motor-associated cortex, thalamus, and cerebellum, thereby suggesting that structures influenced by STN-DBS are partly associated with areas involved in depression [36,37]. In contrast, studies have shown that changes in glucose metabolism induced by STN-DBS affect the levels of ketamine, which has rapid antidepressant and anti-anhedonic effects in brain structures [38,39]. This finding may provide a reasonable explanation for why STN-DBS eases depressive symptoms in PD.

Some previous studies have concluded that, contrary to our findings, STN-DBS worsens depressive symptoms in patients with PD. Therefore, we further investigated the possible reasons for these disparate results. First, the different scales used across studies focus on different sub-symptoms of depression, and the symptoms improved by STN-DBS might differ from the symptoms focused on in other scales that have been used [40]. Research conducted by Strutt and colleagues, using the Beck Depression Inventory scale, which focuses on cognitive-emotional symptoms (self-disappointment and self-criticalness) instead of somatic symptoms of depression, has shown an increase in depression postoperatively [41]. In addition, evidence has already indicated that STN-DBS can enable the dosage of levodopa medication to be decreased [29]. A rapid decrease in levodopa medication has been suggested to cause the deterioration of depressive symptoms [42].

Moreover, studies have concluded that major depressive disorder is characterized by different involvement of the two hemispheres; however, these results require further research [43,44]. In the initial stage of Parkinson’s disease, symptoms appeared on the left or right side of the body. Different sides of the initial symptoms of the disease could have resulted from abnormalities in different cerebral hemispheres. A lot of PD patients have more severe symptoms in one limb than the other. This may also relate to different hemispheres. Our study focused on the changes in the whole brain induced by STN-DBS, rather than unilateral hemispheres. Further attention should be paid to the effect of each unilateral cerebral hemisphere on depression. By exploring the effects of STN-DBS in ameliorating depressive symptoms and predicting the efficacy of STN-DBS in treating depression in patients with PD, our study may help clinicians and nurses to arrange clinical treatment methods according to the status of each patient’s illness. In addition, this study may provide a reference for decision-making and the development of individualized treatment plans, and may help to alleviate patient stress.

Several limitations of our study should be noted. First, the number of patients enrolled in this experiment was 118; thus, the sample size was relatively limited. More patient information must be collected to improve the data statistics. Simultaneously, most participants in the experiment were followed up for no more than 2 years; thus, the data may not reflect the depression of patients with STN-DBS after longer periods of time and consequently might have resulted in inaccurate conclusions. Therefore, a longer follow-up period is necessary. Second, as described above, some studies have suggested the differing involvement of the two hemispheres in depression, but our study focused on the effects on the whole brain. Thus, further attention should be paid to the differences between hemispheres through separate analysis. Third, different scales were used to assess patients’ motor function, namely, UPDRS and MDS-UPDRS, because of differences in the timing of patients’ assessments. Although we used a validated formula to convert the total scores between scales, the results of our study might possibly have been influenced by this. Finally, we only recorded whether the patients were taking antidepressant drugs and we were unaware of the types and dosages of antidepressants that patients used. In future studies, we will keep more detailed records of the antidepressant usage of PD patients with depression.

## 5. Conclusions

In this study, we concluded that STN-DBS could improve depressive symptoms in patients with Parkinson’s disease. We innovatively divided the PD patients into two groups according to their levels of depression (group I and group Ⅱ) and found that patients with more severe depression preoperatively tended to have better improvements after STN-DBS. Furthermore, we performed logistic analysis and found that preoperative depression and gender were predictive factors of postoperative depression outcomes. Our findings could help clinicians to better manage depressive symptoms in PD patients with depression. Follow-up studies should pay more attention to functional imaging or electrophysiology methods in order discover the specific neural mechanisms underlying these findings.

## Figures and Tables

**Table 1:** Preoperative baseline characteristics of patients in Group I and Group II.

	Group I (*n* = 78)	Group II (*n* = 40)	*p*-Value
Gender/Female (%) ^a^	29 (37.18%)	24 (60.00%)	0.018 *
Age of onset(years)	49.91 ± 8.80	49.75 ± 9.84	0.928
Duration(years)	12.41 ± 3.99	13.50 ± 3.89	0.145
Age of surgery (years)	62.32 ± 8.52	63.25 ± 9.00	0.580
LEDD (mg)	673.10 ± 336.72	727.03 ± 389.72	0.354
Pre-Antidepressants ^a^	9 (11.54%)	10 (32.5%)	0.060
Hoehn–Yahr Stage (med off)	2.95 ± 0.52	3.05 ± 0.52	0.111
HAM-D_24_ Score	14.44 ± 3.09	26.18 ± 3.83	<0.001 *
HAMA Score	14.09 ± 5.63	23.14 ± 7.75	<0.001 *
MDS-UPDRS-Ⅲ (med off)	54.87 ± 17.63	58.80 ± 20.54	0.281
MMSE	27.67 ± 1.92	27.15 ± 1.92	0.152
PDQ-39	51.12 ± 17.75	73.23 ± 19.90	<0.001 *
Follow-up (months)	9.78 ± 1.77	9.88 ± 1.68	0.750

Continuous variables are presented as mean ± SD and categorical variables are presented as percentages. *p*-values < 0.05 were considered to be significant and are marked with *. ^a^ Chi-squared tests were applied, and unindicated comparisons were conducted using independent *t*-tests. Abbreviations: Group I: PD patients with mild depression. Group II: PD patients with moderate depression. LEDD: levodopa equivalent daily dose, MDS-UPDRS-Ⅲ: Movement Disorder Society Unified Parkinson’s Disease Rating Scale part Ⅲ, Med off: off medication, HAMA: Hamilton Rating Scale for Anxiety, HAM-D_24_: 24-item Hamilton Depression Rating Scale, PDQ-39: Parkinson’s Disease Questionnaire, MMSE: Mini-Mental State Examination.

**Table 2:** Comparison of preoperative and postoperative HAM-D_24_ total scores and subitems between groups.

	Group I (*n*= 78)			Group II (*n*= 40)			Interaction Effect *p*-Value
	Preoperative	Postoperative	Post Hoc *p-*Value	Preoperative	Postoperative	Post Hoc *p*-Value	
HAM-D_24_-Total score	14.44 ± 3.09	10.99 ± 6.32	<0.001 *	26.18 ± 3.83	17.45 ± 9.23	<0.001 *	<0.001 *
Anxiety/Somatization	3.38 ± 1.61	2.54 ± 2.38	0.020 *	5.90 ± 1.69	3.85 ± 2.43	<0.001 *	0.027 *
Weight Loss	0.10 ± 0.41	0.04 ± 0.19	0.620	0.27 ± 0.55	0.12 ± 0.40	0.181	0.428
Cognitive Disturbances	2.05 ± 1.61	1.73 ± 1.60	0.603	5.10 ± 2.57	3.05 ± 3.21	<0.001 *	0.001 *
Circadian Fluctuations	0.68 ± 0.69	0.37 ± 0.58	0.002 *	0.75 ± 0.74	0.42 ± 0.54	0.026 *	0.913
Retardation Symptoms	2.19 ± 1.36	1.56 ± 1.23	0.012 *	4.27 ± 1.44	2.97 ± 2.18	<0.001 *	0.083
Sleep Disturbances	3.01 ± 1.96	2.38 ± 2.08	0.030 *	4.15 ± 1.62	2.85 ± 1.73	<0.001 *	0.126
Hopelessness Symptoms	3.01 ± 1.51	2.36 ± 1.51	0.016 *	5.72 ± 1.58	4.17 ± 1.92	<0.001 *	0.033 *

All data were presented as mean ± SD. *p*-values < 0.05 were considered to be significant and are marked with * Two-way repeated-measures ANOVA, with group (group I and group II) as the between-group factor and time (before and after DBS) as the within-group factor, was applied to analyze the differences in the HAM-D_24_ total scores and HAM-D_24_ subitems, followed by Bonferroni’s post hoc tests. Anxiety/somatization (6 items: psychic anxiety, somatic anxiety, gastrointestinal symptoms, hypochondriasis, insight, and general symptoms); weight loss (1 item); cognitive disturbances (6 items: self-guilt, suicide, agitation, depersonalization and derealization, paranoid, and obsessive-compulsive symptom); circadian fluctuations (1 item); retardation symptoms (4 items: depression, work and interests, retardation, and sexual symptoms); sleep disturbances (3 items: difficulty falling asleep, superficial sleep and early awakening); hopelessness symptoms (3 items: helplessness, hopelessness and worthlessness). Abbreviations: Group I: PD patients with mild depression. Group II: PD patients with moderate depression. HAM-D_24_: 24-item Hamilton depression rating scale.

**Table 3:** Change of depressive symptoms, motor, cognition and quality of life after STN-DBS between groups.

	Group I (*n*= 78)	Group II (*n*= 40)	Independent *t*-test *p*-Value	ANCOVA *p*-Value
Change of HAM-D_24_ Total score	3.45 ± 6.22	8.73 ± 9.96	0.003 *	0.002 *
Change of HAM-D_24_ level ^a^			0.002 *	*n*/A
Improved	31 (39.74%)	28 (70.00%)		
Not Improved	47 (60.26%)	12 (30.00%)		
Change of HAM-D_24_ subitems				
Anxiety/Somatization	0.85 ± 2.56	2.05 ± 3.13	0.027 *	0.113
Weight Loss	0.06 ± 0.47	0.15 ± 0.70	0.486	0.108
Cognitive Disturbances	0.32 ± 1.93	2.05 ± 3.84	0.010 *	0.026 *
Circadian Fluctuations	0.31 ± 0.83	0.33 ± 0.80	0.913	0.832
Retardation Symptoms	0.63 ± 1.77	1.30 ± 2.32	0.083	0.144
Sleep Disturbances	0.63 ± 2.39	1.30 ± 1.92	0.127	0.064
Hopelessness Symptoms	0.65 ± 2.13	1.55 ± 2.15	0.033 *	0.018 *
MDS-UPDRS-Ⅲ-improvement	49.63 ± 33.11%	54.18 ± 30.87%	0.768	0.777
Change of MMSE score	1.36 ± 3.62	0.68 ± 2.97	0.305	0.287
Change of PDQ-39 score	12.95 ± 24.39	19.93 ± 8.46	0.168	0.550
Antidepressants ^a^	4 (6.41%)	6 (17.50%)	0.140	N/A

Continuous variables are presented as mean ± SD and categorical variables are presented as percentages. ^a^ chi-square test, unindicated comparisons were conducted using independent *t*-test and ANCOVA adjusting for covariates of gender and preoperative HAMA scores. *p*-value < 0.05 is considered to be significant and is marked with * Anxiety/somatization (6 items: psychic anxiety, somatic anxiety, gastrointestinal symptoms, hypochondriasis, insight, and general symptoms); Weight loss (1 item); Cognitive disturbances (6 items: self-guilt, suicide, agitation, depersonalization and derealization, paranoid, and obsessive-compulsive symptom); Circadian fluctuations (1 item); Retardation symptoms (4 items: depression, work and interests, retardation, and sexual symptoms); Sleep disturbances (3 items: difficulty falling asleep, superficial sleep and early awakening); Hopelessness symptoms (3 items: helplessness, hopelessness and worthlessness). Abbreviations: Group I: PD patients with mild depression. Group II: PD patients with moderate depression; HAM-D24, 24-item Hamilton depression rating scale; MDS-UPDRS-Ⅲ, Movement Disorder Society Unified Parkinson’s Disease Rating Scale part Ⅲ; HAMA, Hamilton Rating Scale for Anxiety; PDQ-39, Parkinson’s Disease Questionnaire.

**Table 4:** Relative change and effective size of HAM-D_24_ subitems in group I and group II.

	Relative Change		Effective Size(95%CI)		Classification	
	Group I	Group II	Group I	Group II	Group I	Group II
Anxiety/Somatization	−0.25	−0.35	0.33 (0.10, 0.56)	0.66 (0.31, 0.99)	small	medium
Weight Loss	−0.60	−0.54	0.14(−0.09, 0.36)	0.21 (−0.10, 0.53)	-	small
Cognitive Disturbances	−0.16	−0.40	0.17 (−0.06, 0.39)	0.53 (0.20, 0.86)	-	medium
Circadian Fluctuations	−0.46	−0.44	0.37 (0.14, 0.60)	0.41 (0.08, 0.73)	small	small
Retardation Symptoms	−0.29	−0.30	0.35 (0.12, 0.58)	0.56 (0.22, 0.89)	small	medium
Sleep Disturbances	−0.21	−0.31	0.26 (0.04, 0.49)	0.68 (0.33, 1.01)	small	medium
Hopelessness Symptoms	−0.22	−0.27	0.31 (0.08, 0.53)	0.72 (0.37, 1.07)	small	medium

Relative Change = (mean follow-up-mean baseline)/mean baseline. Effective Size = (mean baseline-mean follow-up)/SD change score. ‘small’ (0.20–0.49) ‘medium’ (0.50–0.79). Anxiety/somatization (6 items: psychic anxiety, somatic anxiety, gastrointestinal symptoms, hypochondriasis, insight, and general symptoms); Weight loss (1 item); Cognitive disturbances (6 items: self-guilt, suicide, agitation, depersonalization and derealization, paranoid, and obsessive-compulsive symptom); Circadian fluctuations (1 item); Retardation symptoms (4 items: depression, work and interests, retardation, and sexual symptoms); Sleep disturbances (3 items: difficulty falling asleep, superficial sleep and early awakening); Hopelessness symptoms (3 items: helplessness, hopelessness and worthlessness). Abbreviations: Group I: PD patients with mild depression. Group II: PD patients with moderate depression.

**Table 5:** Possible predictors of efficacy of STN-DBS on Parkinson’s disease depression.

	Univariable		Multivariable	
	β/OR (95%CI)	*p*-Value	β/OR (95%CI)	*p*-Value
Age	0.99 (0.94, 1.03)	0.488		
Gender				
Male	1.00	−	1.00	-
Female	0.54 (0.26, 1.12)	0.097 *	0.35 (0.15, 0.81)	0.014 *
Age of onset	0.98 (0.94, 1.02)	0.401		
Pre-LEDD	1.00 (1.00, 1.00)	0.190		
Pre-MDS-UPDRS-Ⅲ (med off)	1.01 (0.99, 1.03)	0.518		
Pre-HAMA	1.02 (0.97, 1.07)	0.384		
Pre- HAM-D_24_ level				
Mild Depression	1.00	−	1.00	
Moderate Depression	3.54 (1.57, 7.99)	<0.002 *	4.92 (2.00, 12.13)	<0.001 *
Pre-PDQ-39	1.01 (0.99, 1.03)	0.185		
Pre-Hoehn-Yahr Stage	1.49 (0.71, 3.11)	0.288		
Pre-MMSE	1.17 (0.97, 1.42)	0.105		
Duration	1.02 (0.93, 1.12)	0.675		

*p*-value < 0.05 is considered to be significant and is marked with *. Univariable regression and Multivariable regression were applied to seek possible predictors. Abbreviations: LEDD, Levodopa equivalent daily dose; MDS-UPDRS-Ⅲ, Movement Disorder Society Unified Parkinson’s Disease Rating Scale part Ⅲ; Med off, medication off; HAMA, Hamilton Rating Scale for Anxiety; HAM-D_24_, 24-item Hamilton Depression Rating Scale; PDQ-39, Parkinson’s Disease Questionnaire; MMSE, Mini-Mental State Examination.

## Data Availability

The data presented in this study are available on request from the corresponding author. The data are not publicly available due to privacy regulations for patients.
